# A comparison of trends in melanoma mortality in New Zealand and Australia: the two countries with the highest melanoma incidence and mortality in the world

**DOI:** 10.1186/1471-2407-13-372

**Published:** 2013-08-06

**Authors:** Mary Jane Sneyd, Brian Cox

**Affiliations:** 1 Hugh Adam Cancer Epidemiology Unit, Dunedin School of Medicine, University of Otago, Dunedin, New Zealand

**Keywords:** Melanoma, Mortality, Trends

## Abstract

**Background:**

New Zealand and Australia have the highest incidence and mortality rates from cutaneous melanoma in the world. The predominantly fair-skinned New Zealanders and Australians both enjoy sun, tanned skin and the outdoors, and differences in these activities among generations have been important determinants of trends in melanoma mortality.

We examined whether New Zealand trends in melanoma mortality mirror those in Australia, through detailed comparison of the trends in both countries from 1968 to 2007.

**Methods:**

Five-year age-specific and age-standardised mortality rates were calculated for each country for 5-year time periods. Tests for trends in age-specific rates were performed using the Mantel-Haenszel extension chi-square test. The age-adjusted mortality rate ratios for New Zealand/Australia were plotted against period of death to show relative changes in mortality over time. Age-specific mortality rates were plotted against period and the median year of birth to illustrate age-group and birth cohort effects. To compare the mortality of birth cohorts, age-adjusted melanoma mortality rate ratios were calculated for the birth cohorts in the quin-quennial tables of mortality rates.

**Results:**

The age-standardised mortality rate for melanoma increased in both sexes in New Zealand and Australia from 1968 to 2007, but the increase was greater in New Zealanders and women in particular. There was evidence of recent significant decreases in mortality in younger Australians and less so in New Zealand women aged under 45 years. Mortality from melanoma increased in successive generations born from about 1893 to 1918. In Australia, a decline in mortality started for generations born from about 1958 but in New Zealand there is possibly a decrease only in generations born since 1968.

**Conclusions:**

Mortality trends in New Zealand and Australia are discrepant. It is too early to know if the pattern in mortality rates in New Zealand is simply a delayed response to melanoma control activities compared with Australia, whereby we can expect the same downward trend in similar age groups in the next few years. Specific research is needed to better understand and control the increases in mortality and thickness of melanoma in New Zealand.

## Background

New Zealand and Australia have the dubious distinction of having the highest incidence rates of and mortality rates from cutaneous melanoma in the world [1]. In 2007 in New Zealand, from a total population of 4 million people, 2,173 people were registered with melanoma and 292 people died from the disease [2], whereas in Australia, a country of 21 million inhabitants, 10,342 people were diagnosed with melanoma and 1,279 died from it [3].

The major modifiable risk factor for melanoma is exposure of the skin to UV radiation, with total dose determined by both ambient UV and personal behaviour. New Zealand and Australia have high levels of ambient UV radiation. The major Australian cities range in latitude from Darwin (12.3°S) to Hobart (42.5°S), with summer UV indices (where 1 UV Index unit = 25 mW/m^2^) [4] ranging between 15 and 11. New Zealand has a range of latitude from Kaitaia (35°S) to Bluff (46°S), with summer UV indices from 13 to 8. An overall trend of decreasing melanoma incidence with increasing latitude occurs in both countries.

Melanoma incidence and mortality rates have been rising among white populations world-wide for many years [5] stimulating the development of public health policies encouraging reduced exposure to the sun. Promotion of sun-safety messages started in some Australian states in the 1960s [6,7]: the first large-scale programme started in 1980 [8]. In New Zealand the first skin cancer prevention campaign started in the mid-1980s, and in 1987 this changed to a greater emphasis on melanoma specific prevention [9].

The predominantly fair-skinned New Zealanders and Australians share a liking for sun, tanned skin and outdoor activities and it has been suggested that differences in these activities among generations of Australians and New Zealanders have been important determinants of the trends in melanoma mortality [10,11]. In some countries, including Australia, these trends in incidence and mortality may be changing and a decreasing risk of melanoma for more recent generations in some countries has been observed [7,12,13]. For many years New Zealand has relied on importing Australian sun protection activities and Australian skin cancer research to guide its health promotion and policy direction on the assumption that its melanoma trends would mirror those in Australia. To assess the accuracy of this supposition, we conducted a detailed comparison of the melanoma mortality trends in Australia and New Zealand using routinely collected data from 1968 to 2007.

## Methods

The annual numbers of deaths from melanoma from 1968 to 2007 and mean annual total population estimates were obtained from publications of the New Zealand Ministry of Health [14,15] and, for Australia, equivalent data were obtained from the Australian Institute of Health and Welfare (http://www.aihw.gov.au). Registration of death has been compulsory in New Zealand and Australia throughout the time period studied and both countries have coded cause of death to the relevant International Statistical Classification of Diseases and Related Health Problems. In Australia the Australian Institute of Health and Welfare provides reliable, regular and relevant information and statistics on Australia’s health and welfare [3]. In Australia, deaths are registered by the Registrars of Births, Deaths and Marriages in each State and Territory and since 1906 the Bureau of Census and Statistics has compiled the information collected by the Registrars and published national death statistics [16].

As 2007 is the most recent year for which suitably detailed published data for mortality by sex and age-group are available in Australia, data for New Zealand were restricted to the same period. Since death from melanoma is rare in children, only deaths occurring in people aged 15 years or more were included in the analysis.

Statutory notification of pathology reports of cancer in New Zealand was not introduced until 1 July 1994, and the introduction had a major impact on the reporting of melanoma, resulting in insufficient years of complete data available for a similar comparison of melanoma incidence between the two countries. Therefore this paper is restricted to a comparison of melanoma mortality.

Five-year age-specific mortality rates were calculated for both countries over successive 5-year time periods. Age-standardised rates were calculated using Segi’s world standard population [17]. Statistical tests for trends in age-specific rates, tests for the overall trend and non-linearity of any trend were performed using the Mantel-Haenszel extension chi-square test [18]. Age-specific rates for both countries were plotted to show the contour of melanoma mortality over various times and age groups for each sex. The age-adjusted mortality rate ratios for New Zealand/Australia were plotted against period of death to show relative changes in mortality trends over time, and age-specific mortality rates were plotted against period and the median year of birth to illustrate age-group and birth cohort effects [19]. For comparisons of the melanoma mortality experience of birth cohorts between the countries, age-adjusted melanoma mortality rate ratios were calculated over the age groups available for the birth cohorts represented in the quin-quennial tables of mortality rates.

## Results

Between 1968 and 2007, 6,721 New Zealanders and 29,825 Australians died from melanoma. In New Zealand in 2007 the age-standardised mortality rates (ASMR) for melanoma for men and women were 6.4 and 3.4 per 100,000, respectively: from a total population of 4 million people, 178 men and 114 women died from the disease. In Australia in 2007, a country of 21 million inhabitants, 864 men and 415 women died from melanoma with an ASMR of 5.8 per 100,000 for men and 2.5 per 100,000 for women.

**Figure 1:**
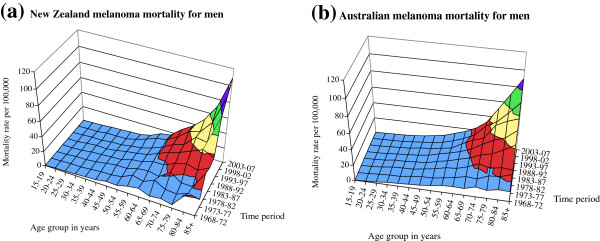
Contour surfaces of male melanoma mortality for (a) New Zealand and (b) Australia.

**Figure 2:**
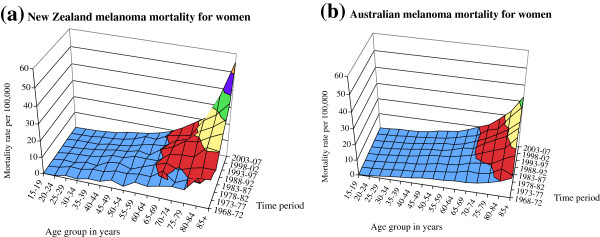
Contour surfaces of female melanoma mortality for (a) New Zealand and (b) Australia.

The contour graphs for age, period of death, and melanoma mortality rate for New Zealand and Australian men are shown in Figure 1 and for women in Figure 2. These represent the surface of melanoma mortality for the population during the years 1968–2007 in five-year age groups. The surfaces demonstrate the pronounced increase in mortality since the 1970s, particularly in older age groups, for both men and women in Australia and New Zealand. Melanoma mortality was higher in New Zealand than in Australia in the most recent years, particularly at older ages, for both men and women. The determinants of mortality that govern these surfaces include age effects (increased mortality with age), generational or cohort effects (experiences of risk or protective factors for the lifetime of each generation included), and period effects (for example, changes in risk factors, stage at presentation, melanoma subtype, or treatments that have occurred across all age groups in particular time periods).

### Comparison by period of death

**Figure 3:**
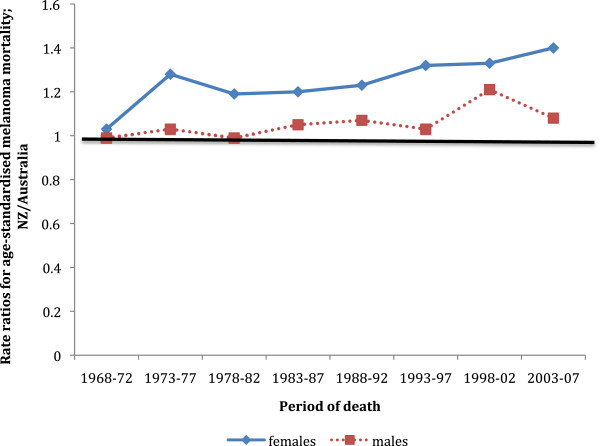
**Rate ratios for age-standardised melanoma mortality: New Zealand/Australia.** The line of equal mortality rates (rate ratio = 1) is marked.

From 1968 to 2007 the age-standardised mortality rate for melanoma increased in both New Zealand and Australian men (see Additional file 1: Table S1), but more so in New Zealand men (Figure 3). During the years 1968–1997 mortality rates for melanoma were very similar in New Zealand and Australian men (age-adjusted mortality rate ratios ranged from 0.99 to 1.07), but in 1998–2002 and 2003–2007 the mortality rates in New Zealand men were significantly higher than in Australian men. Among women the pattern of mortality rates was somewhat different. Although the mortality rates from melanoma increased significantly in both countries after 1968–1972 (see Additional file 1: Table S2), they increased more in New Zealand women and considerably earlier than occurred for men. From 1973–1977 all mortality rates in New Zealand women were significantly higher than Australian women, and by 2003–2007 New Zealand women had a 40% higher melanoma mortality rate compared to Australia (Figure 3).

### Comparisons by period of death and age group

As the time trends in mortality were significantly heterogeneous (p < 0.001) with age group for each sex, the overall age-standardised rates conceal important and divergent age-specific trends in both countries.

**Figure 4:**
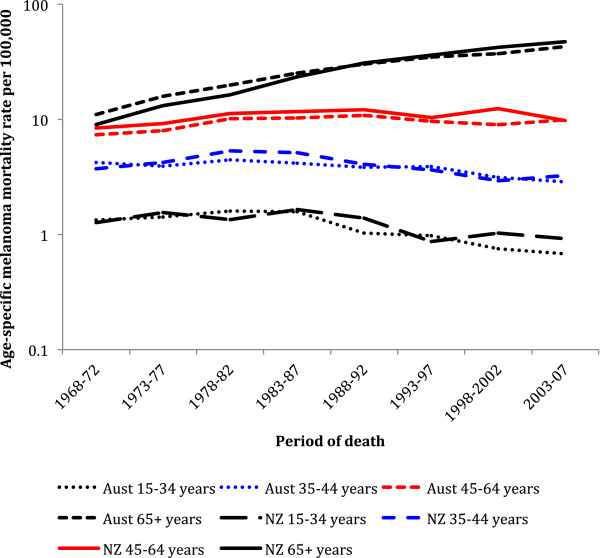
**Trends over time in melanoma mortality for New Zealand and Australian men by age group.** (y-axis on log scale).

In New Zealand from 1968–72 to 2003–07 (Figure 4) no statistically significant reduction in melanoma mortality occurred in men 15–34 years (p = 0.26) or 35–44 years of age (p = 0.88), while over the same time significant increases were observed for men 45–64 years (p < 0.001) and 65 or more years of age (p < 0.001). In contrast in Australia (Figure 4), significant decreases in melanoma mortality occurred in men 15–34 and 35–44 years of age (p < 0.001 for both), whereas mortality significantly increased for men 45 or more years of age (p < 0.001). The trends over time were significantly non-linear for Australian and New Zealand men 45–64 years of age.

**Figure 5:**
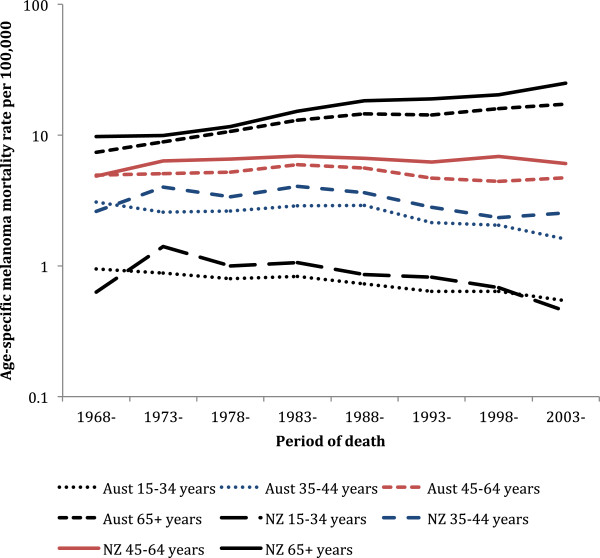
**Trends over time in melanoma mortality for New Zealand and Australian women by age group.** (y-axis on log scale).

From 1968–72 to 2003–07 in New Zealand, melanoma mortality significantly decreased in women 15–34 (p = 0.009) and 35–44 years of age (p = 0.04; Figure 5), with no significant change in women 45–64 years of age, and increased in women 65 or more years of age (p < 0.001). However, in Australia over the same time, female melanoma mortality has decreased significantly in the 3 youngest age groups (15–34 years, p < 0.001; 35–44 years, p < 0.001; and 45–64 years, p = 0.008), but increased significantly for women 65 or more years of age (p < 0.001).

In the 2003–07 time period the increase in melanoma mortality with age for men was very similar in both countries (see Additional file 1: Table S1). Conversely, for women aged 35 or over mortality in New Zealand was higher than in Australia by between 24% and 69%, with statistically significant higher mortality for women aged 40–44, 70–74, and 80 or more years of age (see Additional file 1: Table S2).

### Comparison by birth cohort

**Figure 6:**
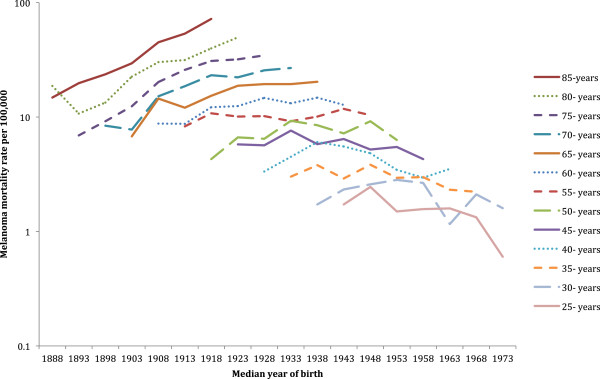
**New Zealand melanoma mortality by 5-year age group and median year of birth.** Both sexes combined. (y-axis on log scale).

**Figure 7:**
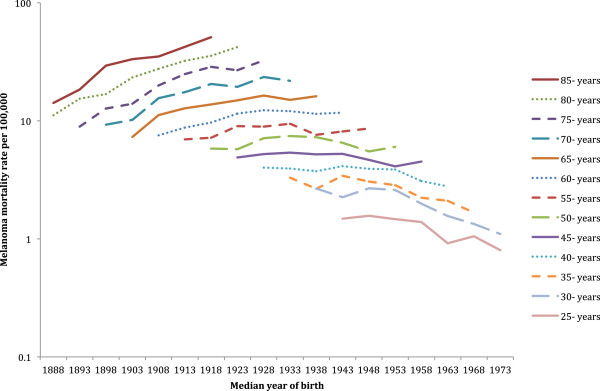
**Australian melanoma mortality by 5-year age group and median year of birth.** Both sexes combined. (y-axis on log scale).

The New Zealand age-specific melanoma mortality rates by birth cohort (identified by their median year of birth) for both sexes combined are shown in Figure 6. Melanoma mortality increased in each generation born from about 1893 until 1918. For New Zealanders born after 1968 there appeared to be a decrease in mortality rates, but death from melanoma was infrequent in these most recent birth cohorts due to their young age. A similar graphical representation of age-specific mortality rates by birth cohort in both sexes combined is shown for Australia in Figure 7. Mortality from melanoma in Australia increased successively for each generation until those born about 1923. Then for people born since 1958, their mortality from melanoma tended to decline.

Male mortality rate ratios for these birth cohorts (see Additional file 1: Table S1) indicated that New Zealand melanoma mortality has been significantly higher than in Australia for men born about 1933 to 1948 and about 1968. New Zealand women born about 1888, 1913 to 1948, and 1958 to 1963 also had significantly higher mortality than their Australian equivalents (see Additional file 1: Table S2). No New Zealand birth cohort for either men or women had significantly lower mortality than the corresponding Australian birth cohort.

## Discussion

The data for this population-based study came from routinely collected and published death statistics for Australia and New Zealand. Melanoma is a common cancer in both countries, and as certification and coding of causes of death are very similar in Australia and New Zealand and follow international guidelines [20,21] a systematic difference in accuracy of cause of death between the two countries is unlikely. Furthermore, systematic differences in determination and recording of cause of death are likely to be small as physicians in New Zealand and Australia are all trained by the Royal Australasian College of Physicians. Under-reporting of melanoma mortality would have to be systematically different for each sex to account for the differences in mortality rates observed, which we consider unlikely.

Similarities in the overall approach to melanoma control in New Zealand and Australia provided an opportunity to assess the relative impact of its implementation. In this population-based study of mortality from melanoma it was shown that although the age-standardised mortality rate for melanoma had increased in both sexes in New Zealand and Australia from 1968 to 2007, the increase in mortality was greater in New Zealanders and New Zealand women in particular. Within these overall increases there was evidence of recent decreases in mortality in younger Australian men and women, and to a lesser degree in New Zealand women aged less than 45 years. Examination of the cohort curves suggested that the increasing mortality in each age group over time was not an effect due to increased age alone, but was also affected by the ageing of generations with higher mortality rates throughout their lives.

It has been previously shown that in New Zealand, overall melanoma mortality increased consistently from 1949 to 1989 predominantly due to birth cohort effects [22]. In women the rates increased approximately linearly over this time whereas in men the rates increased much more rapidly in the second half of the period. An Australian study of annual melanoma mortality diagnosed from 1931 to 2002 found that mortality rates peaked about 1985, significantly decreased after 1985 in people aged under 55 years and stabilized at 55–79 years of age [23]. In many geographic regions with intermediate or low rates of melanoma, such as England and parts of Europe, mortality from melanoma has begun to level off or decline in more recent birth cohorts but this is not universal [24-27]. In Scotland, mortality in young people is still increasing [28] and melanoma mortality in younger US men also increased from 1990 to 2007 [29].

The prevention of death from melanoma is not as effective in New Zealand as in Australia, but the reasons for this are unknown. It is too early to know if the pattern in mortality rates in New Zealand is simply a delayed response compared with Australia whereby we can expect the same downward trend in similar age groups in the next few years, or if New Zealand’s melanoma mortality is on a different trajectory, or a combination of both. Although there is some evidence that mortality in New Zealanders is decreasing in recent generations, men and women born from 1963 to 1968 still experience significantly higher mortality in New Zealand than Australia and it is important to investigate the possible reasons for this.

As survival and mortality from melanoma depend predominantly on thickness at diagnosis (the treatment for melanoma having remained unchanged for many years), the differences in mortality between Australia and New Zealand could be due to differences in the thickness of melanomas at diagnosis: the thickness of melanoma at diagnosis has been increasing in New Zealand for more than a decade [30]. For this to occur, either the presentation of melanoma is different, or techniques have been used in the past that allow earlier diagnosis in Australia, or in New Zealand there is greater delay prior to diagnosis and/or treatment, or the distribution of melanoma subtypes (with dissimilar depth distributions) differ between the two countries.

Improving early detection is an obvious way forward and is likely to result in reductions in mortality more quickly than preventive campaigns [31]. It is possible that the New Zealand public has not recognised suspicious skin lesions as well as Australians and therefore not presented them to health services early enough in their natural history. Approximately half the melanomas detected in Queensland (Australia) and New Zealand were first noticed by the patient and the reported signs and symptoms were very similar [32,33]. For layperson-detected melanomas, over half were noticed first because of a change in colour followed by a change in size, and although itchiness is not a major warning sign of melanoma, 13.2% of patients in Australia and 12.4% in New Zealand said that their lesion was itchy. New Zealanders reported slightly fewer pale or colourless lesions than in Australia (3.5% and 5.3%, respectively) so the proportion of amelanotic lesions, which are less likely to be recognized as suspicious of melanoma, has not contributed to the poorer mortality rates in New Zealand.

Another possibility is that clinicians in Australia diagnosed melanoma earlier than in New Zealand resulting in a better prognostic depth distribution. However, historical evidence does not support this: national Australian data for 1990 to 2006 showed that 62.7% of invasive melanomas diagnosed were thin (<=1.0 mm) and 5.1% were thick (>4.0 mm) [34], similarly, in New Zealand from 1996–2007 63.6% were thin and 7.3% were thick. Furthermore, from 1990–2006 Australian melanoma incidence rates increased in all thickness categories. In the early 1990s in Australia, the increase in thin melanomas was approximately double that of thick melanomas but these increasing trends in thin melanomas have plateaued since 1996. In New Zealand the rate of increase in incidence was greatest in melanomas >1 mm thick and since 1996 the median melanoma thickness has increased [30]. The degree to which these trends are due to delays in diagnosis and treatment are unknown. Internationally no association has been found between the thickest tumours and patient or physician delay, [35-38] rather, it is probable that the greater thickness can be accounted for by more aggressive, rapidly growing lesions [30].

Melanoma subtypes are known to have widely varying depth distributions, with nodular and acral melanomas usually being thickest at diagnosis. In 2000–2003 in Queensland Australia, nodular melanoma accounted for 8% of all invasive melanomas but 38% of melanomas thicker than 2.0 mm [39]. In New Zealand between 1996 and 2007 nodular melanoma contributed 9.8% of all invasive melanomas but only 26.8% of melanomas thicker than 2.0 mm: the greatest percentage of melanomas >2 mm thick (45.1%) were classified as melanoma with no morphology recorded.

High skin cancer incidence and mortality rates in Australia and New Zealand have stimulated the development of public health policies for skin cancer prevention which aim to encourage decreased exposure to sun through a number of personal protective and avoidance measures [8]. The first large-scale sun safety programme ‘Slip! Slop! Slap!’ started in Australia in 1980 [8] followed by SunSmart from 1988 [6]. For about 30 years the programmes have been hosted by a stable and supportive organization and had adequate and reliable funding, without which it is difficult to sustain momentum for a lasting impact [6]. In New Zealand the first skin cancer prevention campaign, also ‘Slip! Slop! Slap!’ started in the mid-1980s but this changed in 1987 to a more focused message on melanoma prevention [9]. It is difficult to determine the impact of these campaigns on melanoma incidence, but any postulated beneficial effect on melanoma mortality has not been pronounced. Without substantial improvements in treatment, improvement in mortality relies on a decrease in the incidence of thick melanomas, not just thin melanomas. However, thus far, the incidence of thick melanomas has not decreased in New Zealand and any possible beneficial effect would appear to have been greater in Australia.

## Conclusions

The points raised highlight the need for the collection of more detailed and accurate data on melanoma and much more research to explain the differing mortality trends found between the Australian and New Zealand populations. Specifically, future research should address the following questions: Is the diagnosis of melanoma made earlier in the natural history of the disease in Australia? Is the stage and thickness at presentation of melanoma subtypes later in New Zealand? Are subtype distributions contributing to differences in mortality? This study also identifies a need to expand New Zealand-based research in order to achieve greater understanding and subsequent control of the increases in mortality and thickness of melanoma in New Zealand.

## Competing interests

The authors declare that they have no competing interests.

## Authors’ contributions

MJS and BC conceived and designed the study. MJS interpreted the data, drafted the manuscript and revised it for intellectual content. BC carried out the analysis and revised the paper for intellectual content. Both authors read and approved the final manuscript.

## Pre-publication history

The pre-publication history for this paper can be accessed here:

http://www.biomedcentral.com/1471-2407/13/372/prepub

## Supplementary Material

Additional file 1: Table S1 Melanoma age-standardised mortality rates and rate ratios for New Zealand and Australian men. **Table S2.** Melanoma age-standardised mortality rates and rate ratios for New Zealand and Australian women. Click here for file
