# Supplementary material for: A comparison of trends in melanoma mortality in New Zealand and Australia: the two countries with the highest melanoma incidence and mortality in the world
Source: BMC Cancer. 2013 Aug 6;13:372. doi: 10.1186/1471-2407-13-372 (PMC3750694; doi:10.1186/1471-2407-13-372)
Supplement: Additional file 1: Table S1 — Melanoma age-standardised mortality rates and rate ratios for New Zealand and Australian men. Table S2. Melanoma age-standardised mortality rates and rate ratios for New Zealand and Australian women. [file 1471-2407-13-372-S1.docx]

Table 1. Melanoma age-standardised mortality rates and rate ratios for New Zealand and Australian men.

| **Male** | New Zealand | | Australia | |  |  |
| --- | --- | --- | --- | --- | --- | --- |
| Time period | n | ASR/100,000 | n | ASR/100,000 | NZ/Aust RR | 95% CI |
| 1968-72 | 216 | 4.45 | 1012 | 4.47 | 0.99 | 0.86-1.15 |
| 1973-77 | 281 | 5.30 | 1285 | 5.14 | 1.03 | 0.91-1.18 |
| 1978-82 | 352 | 6.33 | 1725 | 6.20 | 0.99 | 0.88-1.11 |
| 1983-87 | 441 | 7.07 | 2097 | 6.67 | 1.05 | 0.95-1.16 |
| 1988-92 | 519 | 7.63 | 2527 | 7.05 | 1.07 | 0.98-1.18 |
| 1993-97 | 580 | 7.49 | 2950 | 7.17 | 1.03 | 0.94-1.13 |
| 1998-02 | 749 | 8.43 | 3287 | 6.88 | 1.21 | **1.11-1.30** |
| 2003-07 | 833 | 7.98 | 4097 | 7.30 | 1.08 | **1.01-1.17** |
| Age group (years) | n | Rate per 100,000 2003-07 | n | Rate per 100,000 2003-07 | NZ/Aust RR | 95% CI |
| 15-19 | 0 | 0.00 | 4 | 0.11 | - | - |
| 20-24 | 4 | 0.55 | 13 | 0.36 | 1.54 | 0.51-4.67 |
| 25-29 | 7 | 1.11 | 33 | 0.94 | 1.17 | 0.52-2.65 |
| 30-34 | 15 | 2.19 | 48 | 1.28 | 1.70 | 0.96-3.02 |
| 35-39 | 20 | 2.72 | 80 | 2.15 | 1.27 | 0.78-2.07 |
| 40-44 | 29 | 3.76 | 136 | 3.57 | 1.05 | 0.71-1.57 |
| 45-49 | 31 | 4.30 | 204 | 5.62 | 0.77 | 0.53-1.12 |
| 50-54 | 47 | 7.40 | 263 | 7.87 | 0.94 | 0.69-1.28 |
| 55-59 | 80 | 13.95 | 376 | 12.22 | 1.14 | 0.90-1.45 |
| 60-64 | 74 | 16.47 | 391 | 16.32 | 1.01 | 0.79-1.29 |
| 65-69 | 102 | 28.41 | 425 | 22.6 | 1.26 | **1.01-1.56** |
| 70-74 | 108 | 37.47 | 489 | 32.23 | 1.16 | 0.94-1.43 |
| 75-79 | 117 | 50.25 | 631 | 50.84 | 0.99 | 0.81-1.20 |
| 80-84 | 103 | 71.19 | 559 | 70.15 | 1.01 | 0.82-1.25 |
| 85+ | 96 | 109.14 | 444 | 90.03 | 1.21 | 0.97-1.51 |
| Median year of birth | n |  | n |  | NZ/Aust RR | 95% CI |
| 1883 | 7 |  | 13 |  | 1.88 | 0.76 - 4.64 |
| 1888 | 14 |  | 59 |  | 1.04 | 0.58 - 1.86 |
| 1893 | 21 |  | 114 |  | 0.86 | 0.54 - 1.37 |
| 1898 | 37 |  | 285 |  | 0.59 | 0.42 - 0.83 |
| 1903 | 77 |  | 500 |  | 0.70 | 0.55 - 0.89 |
| 1908 | 236 |  | 976 |  | 1.13 | 0.98 - 1.30 |
| 1913 | 309 |  | 1578 |  | 0.98 | 0.87 - 1.10 |
| 1918 | 461 |  | 2210 |  | 1.09 | 0.98 - 1.20 |
| 1923 | 503 |  | 2470 |  | 1.08 | 0.98 - 1.19 |
| 1928 | 501 |  | 2495 |  | 1.05 | 0.95 - 1.15 |
| 1933 | 412 |  | 1893 |  | 1.13 | **1.01 - 1.25** |
| 1938 | 342 |  | 1495 |  | 1.19 | **1.06 - 1.34** |
| 1943 | 295 |  | 1361 |  | 1.14 | **1.01 - 1.30** |
| 1948 | 275 |  | 1229 |  | 1.19 | **1.04 - 1.36** |
| 1953 | 179 |  | 912 |  | 1.02 | 0.87 - 1.20 |
| 1958 | 121 |  | 619 |  | 0.99 | 0.81 - 1.20 |
| 1963 | 82 |  | 378 |  | 1.07 | 0.84 - 1.36 |
| 1968 | 57 |  | 200 |  | 1.44 | **1.07 - 1.93** |
| 1973 | 23 |  | 103 |  | 1.20 | 0.76 - 1.88 |
| 1978 | 12 |  | 60 |  | 1.06 | 0.57 - 1.96 |
| 1983 | 4 |  | 17 |  | 1.17 | 0.39 - 3.46 |

Table 2. Melanoma age-standardised mortality rates and rate ratios for New Zealand and Australian women.

| **Female** | New Zealand | | Australia | |  |  |
| --- | --- | --- | --- | --- | --- | --- |
| Time period | n | ASR/100,000 | n | ASR/100,000 | NZ/Aust RR | 95% CI |
| 1968-72 | 166 | 3.02 | 741 | 3.04 | 1.03 | 0.87-1.22 |
| 1973-77 | 234 | 4.09 | 862 | 3.09 | 1.28 | **1.11-1.47** |
| 1978-82 | 252 | 3.99 | 1020 | 3.23 | 1.19 | **1.04-1.37** |
| 1983-87 | 323 | 4.53 | 1324 | 3.68 | 1.2 | **1.07-1.36** |
| 1988-92 | 367 | 4.53 | 1527 | 3.64 | 1.23 | **1.10-1.38** |
| 1993-97 | 398 | 4.28 | 1542 | 3.22 | 1.32 | **1.18-1.47** |
| 1998-02 | 461 | 4.3 | 1780 | 3.18 | 1.33 | **1.20-1.47** |
| 2003-07 | 549 | 4.31 | 2049 | 3.23 | 1.40 | **1.27-1.53** |
| Age group (years) | n | Rate per 100,000. 2003-07 | n | Rate per 100,000. 2003-07 | NZ/Aust RR | 95% CI |
| 15-19 |  | 0 | 0.00 | 1 | 0.03 | - |
| 20-24 | 2 | 0.28 | 10 | 0.28 | 1.00 | 0.22-4.54 |
| 25-29 | 3 | 0.46 | 30 | 0.87 | 0.53 | 0.16-1.70 |
| 30-34 | 8 | 1.07 | 35 | 0.92 | 1.16 | 0.54-2.49 |
| 35-39 | 14 | 1.76 | 45 | 1.19 | 1.48 | 0.81-2.68 |
| 40-44 | 27 | 3.3 | 77 | 1.99 | 1.66 | **1.07-2.56** |
| 45-49 | 32 | 4.28 | 127 | 3.44 | 1.24 | 0.84-1.83 |
| 50-54 | 34 | 5.24 | 142 | 4.21 | 1.25 | 0.86-1.81 |
| 55-59 | 40 | 6.87 | 152 | 4.98 | 1.38 | 0.98-1.95 |
| 60-64 | 42 | 9.1 | 169 | 7.12 | 1.28 | 0.91-1.79 |
| 65-69 | 48 | 12.65 | 189 | 9.8 | 1.29 | 0.94-1.77 |
| 70-74 | 54 | 17.09 | 201 | 12.24 | 1.40 | **1.04-1.88** |
| 75-79 | 60 | 21.52 | 258 | 17.26 | 1.25 | 0.94-1.65 |
| 80-84 | 77 | 35.4 | 269 | 23.03 | 1.54 | **1.20-1.98** |
| 85+ | 108 | 55.4 | 343 | 32.77 | 1.69 | **1.37-2.09** |
| Median year of birth | n |  | n |  | NZ/Aust RR | 95% CI |
| 1883 | 9 |  | 28 |  | 1.33 | 0.63-2.81 |
| 1888 | 24 |  | 64 |  | 1.70 | **1.07-2.71** |
| 1893 | 29 |  | 168 |  | 0.82 | 0.56-1.22 |
| 1898 | 62 |  | 291 |  | 1.02 | 0.77-1.34 |
| 1903 | 104 |  | 445 |  | 1.10 | 0.89-1.36 |
| 1908 | 173 |  | 715 |  | 1.14 | 0.96-1.34 |
| 1913 | 262 |  | 1054 |  | 1.22 | **1.07-1.40** |
| 1918 | 367 |  | 1335 |  | 1.38 | **1.23-1.55** |
| 1923 | 323 |  | 1232 |  | 1.36 | **1.21-1.54** |
| 1928 | 256 |  | 1134 |  | 1.18 | **1.03-1.35** |
| 1933 | 216 |  | 839 |  | 1.31 | **1.13-1.53** |
| 1938 | 209 |  | 758 |  | 1.38 | **1.19-1.61** |
| 1943 | 178 |  | 714 |  | 1.26 | **1.07-1.49** |
| 1948 | 182 |  | 650 |  | 1.45 | **1.23-1.71** |
| 1953 | 122 |  | 520 |  | 1.21 | 0.99-1.47 |
| 1958 | 102 |  | 384 |  | 1.31 | **1.05-1.63** |
| 1963 | 64 |  | 225 |  | 1.35 | **1.03-1.79** |
| 1968 | 36 |  | 145 |  | 1.19 | 0.83-1.72 |
| 1973 | 19 |  | 74 |  | 1.30 | 0.79-2.15 |
| 1978 | 7 |  | 47 |  | 0.76 | 0.35-1.69 |
| 1983 | 3 |  | 13 |  | 1.14 | 0.32-3.99 |
